# Pros and Cons of Marijuana in Treatment of Parkinson’s Disease

**DOI:** 10.7759/cureus.4813

**Published:** 2019-06-03

**Authors:** Rikinkumar S Patel, Saher Kamil, Mansi R Shah, Narmada Neerja Bhimanadham, Sundus Imran

**Affiliations:** 1 Psychiatry, Griffin Memorial Hospital, Norman, USA; 2 Psychiatry, Austin Hospital, Austin, USA; 3 Psychiatry, Northwell Zucker Hillside Hospital, Glen Oaks, USA; 4 Public Administration / Health Care Administration, Drake University, Des Moines, USA; 5 Neurology, Indiana University School of Medicine, Indianapolis, USA

**Keywords:** medical marijuana, marijuana, parkinson disease, parkinson

## Abstract

Parkinson’s disease (PD) is the second most common neurodegenerative disorder of adult onset in the United States. It is a debilitating condition and presents with both motor and non-motor symptoms. Current treatment options are scarce and include replacement of dopamine deficiency with levodopa which targets only motor symptoms of the disorder, does not halt its progression, and is associated with side effects of its own, including dyskinesia. With medical marijuana gaining popularity and being legalized in the United States, we examined the pros and cons of marijuana in the treatment of Parkinson’s disease.

## Introduction and background

Parkinson’s disease (PD) affects one to two per 1000 individuals, and its prevalence increases with age, affecting 1% of the population above 60 years of age [1]. It is estimated that the number of individuals with PD is expected to increase to nine million by 2030 in fifteen of the most populous countries worldwide [2]. About 3% to 5% of the world’s population is known to use marijuana, making it the most popular illicit substance [3].

In the United States, about 20 million people are marijuana users, estimated to 12.3% of its total population, with about 1.2 million individuals using medical marijuana. As many as 23 states, as well as District of Columbia (DC), have legalized medical marijuana, and four states have legalized its recreational use [3]. The use of marijuana has increased four times in individuals aged 55 to 59 years and doubled among the elderly population aged 60 to 64 years, between 2002 and 2012 [3]. Marijuana is widely used recreationally by older people to self-medicate symptoms of neurological and psychiatric disorders such as PD, multiple sclerosis (MS), amyotrophic lateral sclerosis, and schizophrenia [3]. About 44% of the population with PD and MS is currently using marijuana [4]. 

Marijuana users report lower levels of disability compared to non-marijuana users [4]. In addition to marijuana, there are various marijuana products composed of treatments such as dronabinol, nabiximols, and nabilone that have been used in people with brain disorders [3]. The goal of this review article was to asses the pros and cons associated with the use of marijuana for short-term or long-term purpose in patients with PD.

## Review

Pathophysiology and treatment of Parkinson’s disease

PD arises as a result of the loss of dopaminergic neurons in the substantia nigra, resulting in the loss of control of voluntary movements, which manifests as tremor, rigidity, and bradykinesia [1]. Other non-motor symptoms are psychosis, cognitive impairment, depression, and anxiety [3]. Its exact cause is unknown, with genetic and environmental factors playing a role to some extent. Current treatment options for PD include activation of dopamine receptors with levodopa, a biosynthetic precursor to dopamine. However, research has indicated that levodopa is only valid during the early and intermediate stages of the disease and causes irreversible dyskinesia in later stages [5]. It provides relief of motor symptoms of PD and does not prevent its progression [2].

Mechanism of action of marijuana

Marijuana consists of 85 phytocannabinoids, from which cannabidiol (CBD) and tetrahydrocannabinol (THC) are the main constituents [2]. CBD and THC are responsible for the medicinal effects of marijuana [2]. CBD is non-psychoactive and is known to have hypnotic, anxiolytic, antipsychotic, and neuroprotective effects [3]. THC is the main component responsible for psychotropic effects of cannabis, and it acts via two types of G protein-coupled receptors, known as cannabinoid type 1 (CB1) and type 2 (CB2) [6]. CB1 receptors are located mainly in the central nervous system (CNS), and CB2 receptors reside in organs and cells of the immune system [7]. The significant presence of cannabinoid receptors in the basal ganglia is the reason behind the significance of cannabis or cannabinoids as possible pharmacotherapy for treating PD and dyskinetic movement disorders [7]. 

While THC is psychoactive and a partial agonist to CB1 and CB2 receptors, CBD is indirectly antagonistic to cannabinoid receptors and modulates the side effects of THC by increasing the CB1-receptor density or some other CB1-mechanism [2]. CBD inhibits the psychotropic effects of THC and improves its tolerability and therapeutic window [2], without being intoxicating [3]. Studies also suggest that cannabinoid antagonists exert anti-parkinsonism effects, while agonists help with motor control [2].

Endocannabinoids are produced in the body and help regulate memory, pleasure, concentration, thinking, movement, concentration, sensory and time perception, appetite, and pain [2]. They act on CB1 and CB2 receptors [2]. The main endocannabinoids are anandamide and 2-arachidonoyl glycerol [2]. Globus pallidus and substantia nigra are major brain areas involved in the control of movements and contain the highest densities of CB1 receptors, as well as the highest levels of endocannabinoids especially anandamide [2]. In a study involving mice, loss of CB1 receptors resulted in decreased locomotor skills [7]. These findings support the importance of the cannabinoids in the control of movement by the basal ganglia [7]. Studies have proposed that dopaminergic D2 receptors regulate anandamide synthesis in the striatum which gives inhibitory feedback to dopamine-induced motor activity [2]. It has been suggested that activation of CB1 receptor neuro-protects against dopaminergic lesions and levodopa-induced dyskinesia [2].

Pros of marijuana in Parkinson’s disease

Medical marijuana has been observed to improve both motor and non-motor symptoms including bradykinesia, rigidity, tremor, sleep, and pain [2]. A study demonstrated the effects of cannabis on 85 individuals with PD. Most of them consumed half a teaspoon of cannabis leaves, along with their prescribed pharmacotherapy for PD. About 46% of these individuals reported relief of PD symptoms in general, occurring at an average of 1.7 months after the first use of marijuana, suggesting chronic use of marijuana may be required for improvement in symptoms. Bradykinesia was the most common symptom that was alleviated among cannabis users, followed by muscle rigidity and tremor. Additionally, 14% of patients reported improvement of levodopa-induced dyskinesia with the use of cannabis [7]. It was also noted that higher urine levels (above 50 ng/ml) of a metabolite of THC in individuals using cannabis for several months resulted in the visible improvement of bradykinesia and rigidity.

THC has shown to improve both activity and hand-eye coordination in an animal-PD model [3]. A clinical study of 22 patients with PD and smoking marijuana, resulted in improvement of motor symptoms such as bradykinesia, resting tremor, rigidity, and posture, along with with non-motor symptoms such as sleep and pain [3]. It has also been observed to improve rapid eye movement (REM) sleep behavior disorder in individuals with PD [3]. Nabilone is a synthetic cannabinoid receptor agonist, and when given with levodopa, it significantly reduces dyskinesia and increases the duration of action of levodopa by 76% [2]. This is in contrast to CB1 antagonist, Rimonabant, which did not produce improvement in motor symptoms in PD [3].

Some people with PD have reported a ‘calming effect’ on their tremor and dyskinesia with the use of marijuana [8]. Another study showed improvement of dyskinesia by up to 30% in PD patients without worsening of symptoms, with CBD withdrawal causing dystonia [2]. A study investigated the use of marijuana and compared it to non-marijuana users in individuals with PD and MS. There was a lower level of disability in marijuana users as compared to non-users. Memory, mood, and fatigue were other factors that cannabis had a positive effect on. About 85% of the patients reported effectiveness as moderate or higher due to improvement in symptoms and no worsening with the use of cannabis [4].

Depression is a common symptom in PD, with a prevalence of up to 50%, that is undiagnosed and undertreated [2]. Endocannabinoids are proposed to regulate mood and behavior, and their loss can lead to depression [2]. It has been demonstrated in epidemiological studies that marijuana users using marijuana daily or weekly exhibit a better mood as compared to non-users, while other studies have shown an association between heavy use of marijuana and depression, but it is unclear whether the depressive symptoms are due to marijuana or other factors [2]. Hence, it is possible that the use of marijuana can help overcome depressive symptoms in PD patients [2]. Also, marijuana has also been proposed to improve sleep, as demonstrated in a clinical trial of 2000 patients with various pain disorders [2]. Studies have further suggested that marijuana can help with spasticity and pain in people with PD and MS and has neuroprotective effects [4]. In Colorado, a survey was conducted for PD patients, and its findings were similar to past results of marijuana alleviating non-motor symptoms [2]. Marijuana not only improves motor and non-motor activities, but also has neuroprotective properties, and helps in improving symptoms of PD as well as delaying its progression. Hence, marijuana can be used as an alternative or add-on option in adults with PD to help improve the overall quality of life patients suffering from PD [2].

Cons of marijuana in Parkinson’s disease

Adverse effects of marijuana include cognitive impairments, although this is temporary and resolves with cessation of the drug. It is well known that marijuana can cause impairment in working memory and may have a positive association with depression [4]. This is contradictory to a study in which individuals with PD who were consuming marijuana had improved memory and mood, which could be due to avoidance of the drug by individuals having problems with memory or mood in fear of worsening of symptoms [4].

Weight gain is another possible side effect of marijuana, as the drug is thought to increase caloric intake [4]. When initiated in adolescence, marijuana can contribute towards obesity but an extensive study of adults demonstrated the lesser prevalence of obesity in marijuana users, and a recent survey of adults with PD and MS did not support obesity in marijuana users [4].

It is suggested that the acute use of marijuana can cause a transient motivational state in marijuana non-users, and regular use prevents it [4]. It is also recommended that marijuana negatively affects motor skills, but in a recent study in PD and MS patients, both users and non-users spent the same amount of time on physical activity and sitting [4].

Short-term Effects of Marijuana

Acute marijuana use impairs consolidation, attention tasks, memory retrieval, verbal memory, executive functions, and learning. Furthermore, frontal dysfunction has also been observed which causes poor planning, decreased information processing speed, lack of self-monitoring, poor planning, and changes in gross and fine motor skills [3].

Long-term Effects of Marijuana

Dependence is the primary concern after long-term use of marijuana. In the US, 10% of adults use marijuana, and one-third of users suffer from abuse or dependence. Furthermore, 30% of marijuana users and 50% to 95% of heavy users experience marijuana withdrawal symptoms. One study followed participants from birth to age 38, and persistent marijuana users had six point reduction in intelligence quotient (IQ) compared to non-users [5]. Chronic marijuana use can result in dependence, cognitive impairments, depression, and anxiety, and increase the risk for lung diseases [3].

## Conclusions

PD is debilitating and can manifest as both motor and non-motor symptoms including bradykinesia, resting tremor, rigidity, and depression. The current treatment provides a cure for the motor symptoms, but only in the initial phase, and has side-effects of its own. Self-medication with marijuana has improved many symptoms including bradykinesia, tremor, rigidity, depression, sleep, and pain. However, the use of marijuana comes with short-term and long-term effects including cognitive problems. It is observed that long-term use of marijuana is needed for its result of taking place, which can also place an individual for risk of the dependence of the illicit drug. More research is required to study the effects of marijuana in patients with PD, for which treatment is limited.
